# Molecular function prediction for a family exhibiting evolutionary tendencies toward substrate specificity swapping: Recurrence of tyrosine aminotransferase activity in the Iα subfamily

**DOI:** 10.1002/prot.24318

**Published:** 2013-05-13

**Authors:** Kathryn E Muratore, Barbara E Engelhardt, John R Srouji, Michael I Jordan, Steven E Brenner, Jack F Kirsch

**Affiliations:** 1 Department of Molecular and Cell Biology, University of CaliforniaBerkeley, California; 2 Department of Electrical Engineering and Computer Science, University of CaliforniaBerkeley, California; 3 Department of Statistics, University of CaliforniaBerkeley, California; 4 Department of Plant and Microbial Biology, University of CaliforniaBerkeley, California; 5 QB3 Institute, University of CaliforniaBerkeley, California

**Keywords:** enzyme, kinetics, phylogenetics, pyridoxal 5'-phosphate, transaminase, aspartate aminotransferase

## Abstract

The subfamily Iα aminotransferases are typically categorized as having narrow specificity toward carboxylic amino acids (AATases), or broad specificity that includes aromatic amino acid substrates (TATases). Because of their general role in central metabolism and, more specifically, their association with liver-related diseases in humans, this subfamily is biologically interesting. The substrate specificities for only a few members of this subfamily have been reported, and the reliable prediction of substrate specificity from protein sequence has remained elusive. In this study, a diverse set of aminotransferases was chosen for characterization based on a scoring system that measures the sequence divergence of the active site. The enzymes that were experimentally characterized include both narrow-specificity AATases and broad-specificity TATases, as well as AATases with broader-specificity and TATases with narrower-specificity than the previously known family members. Molecular function and phylogenetic analyses underscored the complexity of this family's evolution as the TATase function does not follow a single evolutionary thread, but rather appears independently multiple times during the evolution of the subfamily. The additional functional characterizations described in this article, alongside a detailed sequence and phylogenetic analysis, provide some novel clues to understanding the evolutionary mechanisms at work in this family.

## INTRODUCTION

**Scheme 1:**
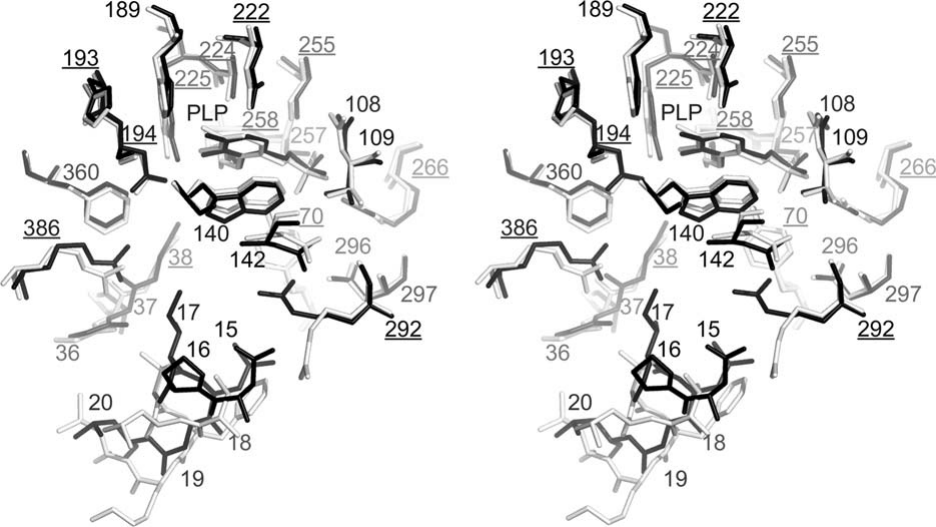
The traditional view of substrate specificity of family Iα aminotransferases. Aspartate aminotransferases (AATases) preferentially catalyze the reversible reaction on the left, while tyrosine aminotransferases (TATases) catalyze both the left and right reversible reactions with comparable rate constants. The α-ketoacids corresponding to the amino acids are oxaloacetate (OAA), α-ketoglutarate (αKG), phenylpyruvate (PP) and hydroxyphenylpyruvate (HPP).

Subfamily Iα aminotransferases are pyridoxal 5′-phosphate (PLP)-dependent enzymes that convert an amino acid into its α-keto acid, with the concomitant synthesis of a second amino acid from its α-keto acid. The primary substrates used by this family of enzymes are aspartate, glutamate, tyrosine, and phenylalanine, and their corresponding keto acids: oxaloacetate (OAA), α-ketoglutarate (αKG), hydroxyphenylpyruvate (HPP), and phenylpyruvate (PP). The extent to which a substrate is preferred varies from enzyme to enzyme. The enzymes have been classified on the basis of this preference into two groups (Scheme 1). Aspartate aminotransferases (AATases) prefer aspartate to the aromatic substrates, while tyrosine aminotransferases (TATases; also known as aromatic aminotransferases) catalyze the transamination of the dicarboxylic and aromatic amino acids with approximately equal rate constants.

Aspartate aminotransferase activity is essential due to its roles in central metabolism. OAA is an intermediate in the citric acid cycle, and Asp is an intermediate for the biosynthesis of other amino acids, nucleotides, and other metabolites. Thus interconversion of Asp and OAA connects these basic processes. In eukaryotes, AATases play a second important role in the malate aspartate shuttle; therefore both mitochondrial and cytosolic isozymes are expressed. While AATases are constitutively expressed in microorganisms such as *Escherichia coli*, TATases are metabolically regulated. In *E. coli*, TATase (eTAT) is used in the biosynthesis of Tyr and Phe as indicated by gene repression by Tyr.^1^ Conversely, the TATase gene in *Pseudomonas aeruginosa* is induced by aromatic amino acids and the enzyme product (PhhC) is used in catabolism of Tyr and Phe.^2^

AATases and TATases perform essential functions, but the AATase and TATase activities can be provided by enzymes within or outside of the Iα subfamily of aminotransferases (such as the mammalian Iγ TATases). Like all members of the Family I and II aminotransferases (Pfam family PF00155^3^), these other aminotransferases share some characteristics with the Iα subfamily aminotransferases. For example, the catalytic base is a lysine residue, which can be aligned across all aminotransferase superfamily sequences, and 11 additional residues are conserved in Family I.^4^ Yet sequence similarity studies have shown the distinct subfamilies to be distinct monophyletic clades in the phylogeny^5^ and kinetic studies have demonstrated some important differences.^6^,^7^ Many organisms possess multiple AATases and TATases in one or more subfamilies, where the redundancy provides more precise functional, temporal, or spatial control over the enzyme activities. Such complexity means that it is not certain, *a priori*, what the substrate specificity of an aminotransferase will be. Nonetheless, the biological data lead to certain inferences; for example, animals tend to have two subfamily Iα AATases—one cytosolic and one mitochondrial, both of which perform functions critical to metabolism—and no TATases from this subfamily.

**Figure 1:**
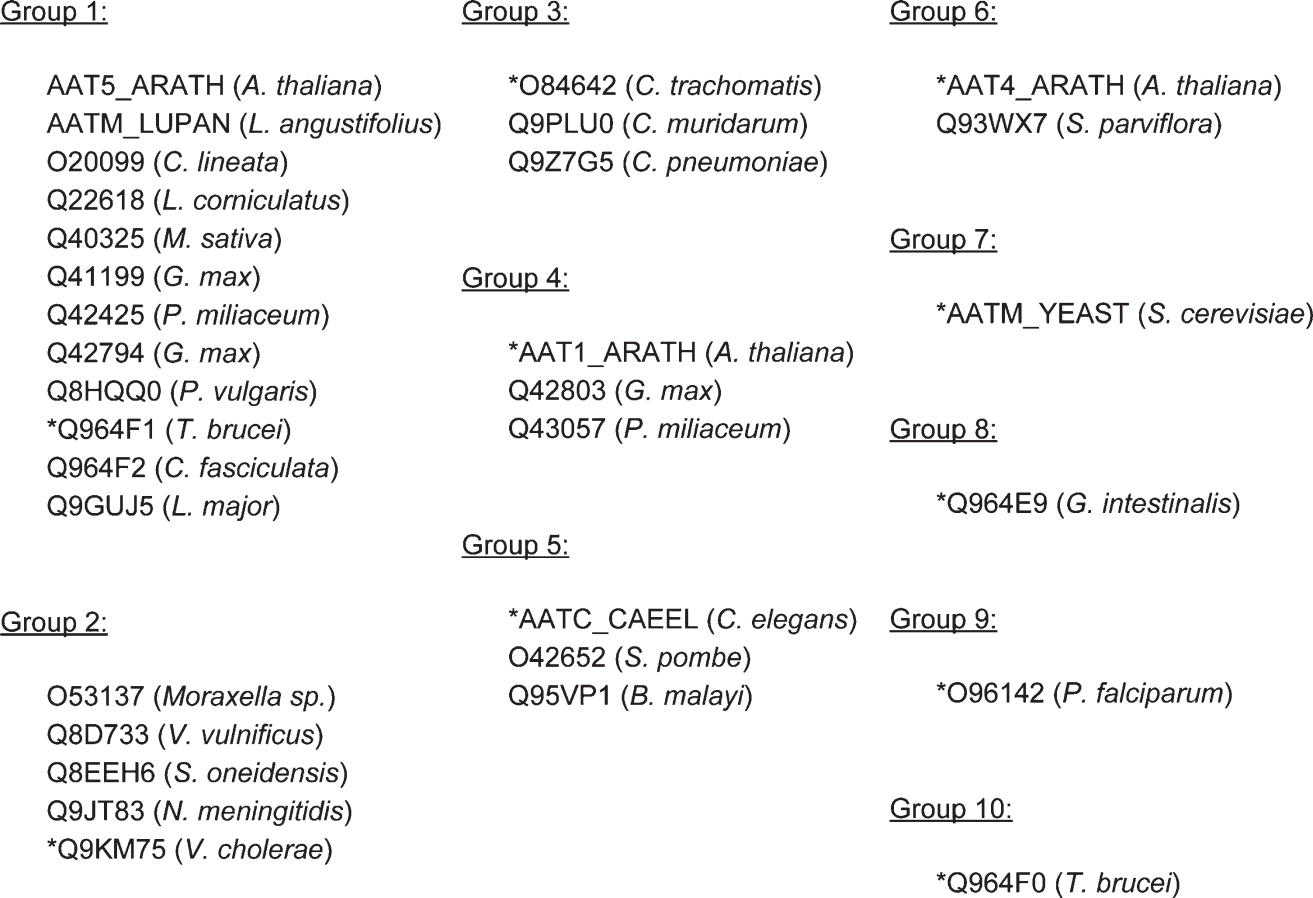
Stereo overlay of subfamily Iα aminotransferases active sites. *E. coli* and pig cytosolic AATase residues are in black and light gray, respectively. The side-chain of the amino acid substrate (not shown) is directed out of the plane, into the pocket of residues at the bottom of each panel. Underlined residues are conserved in the characterized aminotransferase sequences. The PDB codes are 1ASN and 1AJR. This figure was made with PyMOL.^13^

The general molecular function of proteins in sequence databases (such as reaction specificity) is misannotated at a rate of at least 5%,^8^,^9^ while it has been estimated that about one-third of all specific annotations (such as substrate specificity) are incorrect.^9^,^10^ Annotation of the subfamily Iα aminotransferases is no exception, making accurate prediction of substrate specificities of newly sequenced genes within this family challenging.^11^,^12^ The sequences and structures of all enzymes in this subfamily are similar (>30% sequence identity; <1.8 Å r.m.s.d. of C_α_ atoms). Figure 1 shows the nearly superimposable active sites of 2 of the 10 aminotransferases whose crystal structures have been solved.^14^–^21^ With such high sequence and structural similarity, one may hypothesize that the proteins share a similar molecular function and possibly even substrate specificity.^22^

The substrate preference is defined by the ratio of the specificity constants, *k*_cat_/*K*_m_, for each class of substrate. An aminotransferase is an AATase if its ratio for the aspartate reaction to the aromatic reaction is >1. Conversely, a ratio <1 is indicative of a TATase. For example, eAAT has a specificity ratio of 800^23^,^24^ for aspartate to phenylalanine, while eTAT has a specificity ratio of 0.04.^25^ Yet, the sequences of these two enzymes are 42% identical. Furthermore, the PhhC sequence is more similar (46% identity) to that of eAAT than it is to the eTAT sequence (44% identity). Thus, sequence identity is a poor indicator of the substrate specificity within subfamily Iα aminotransferases.

The HEX design, reported by Onuffer and Kirsch, mutated the six known conserved AATase residues (as of 1993) to those found in the eTAT sequence.^26^ The substitutions sufficed to convert eAAT to an enzyme with substantial TATase activity.^26^ The HEX mutations are important in the context of eAAT as the six point mutations do not have identical effects in the presence of other scaffolds. Thus, the context of mutations is a key variable in protein redesign.^23^,^27^ Additionally, there are many solutions to the problem of converting an AATase into a TATase as illustrated by the successful conversion by directed evolution.^28^ These solutions in aggregate challenge our standard models capturing how molecular function evolves and how protein function is controlled by sequence, in that protein function does not appear to evolve in parallel with protein sequence in this subfamily. We would like to generalize these solutions to begin to understand the mechanisms of evolution and function determination. Understanding these mechanisms can ultimately be used to provide more reliable substrate specificity annotations and aid in enzyme design.

The availability of more Iα aminotransferase sequences has revealed more about the subfamily diversity. Some of the enzymes share less than 40% of their amino acid sequence with any other subfamily member with experimentally characterized substrate specificity. The full extent of diversity can be better appreciated if the substrate specificities are known at a higher resolution throughout the family. To this end, a set of diverse aminotransferases was chosen for substrate specificity characterization. We report the kinetic constants for 11 distantly related aminotransferases, and we observed that there are many instances of a single substrate specificity arising independently in the evolutionary history of this protein family. We applied a statistical model for phylogenetic-based molecular function prediction in order to elucidate the evolutionary journey of the different proteins in the aminotransferase family.

## MATERIALS AND METHODS

Reagents were from Sigma-Aldrich (St. Louis, MO) or Fisher (Fairlawn, NJ), unless otherwise indicated.

Malate dehydrogenase (MDH) and hydroxyisocaproate dehydrogenase (HO-HxoDH) were prepared as described previously,^29^,^30^ except that HO-HxoDH was expressed in Rosetta(DE3)pLysS cells (EMD, San Diego, CA) from the plasmid pHicHis described below. The cloning, expression, and purification of aminotransferases are described elsewhere.^31^

### Subcloning of HO-HxoDH

All enzymes used for cloning were from New England Biolabs (Ipswich, MA) except that alkaline phosphatase was obtained from USB (Cleveland, OH). Purification of DNA fragments was carried out using GFX kits from GE Healthcare (Piscataway, NJ).

pHicHis was made by subcloning the HO-HxoDH gene from the pTrc-99a construct, pHicDH-His1, described in Aitken *et al*.,^30^ into pET19b (EMD) to increase expression levels. pHicDH-His1 does not have the unique restriction sites necessary for direct cloning into pET19b, therefore an extra subcloning step was undertaken to introduce a new restriction site. pHicDH-His1 was sequentially digested with NcoI and XbaI restriction enzymes, and the ∼1000 base pair fragment from the pHicDH-His1 digestion was gel purified. This purified fragment was ligated to XbaI-digested pET19b with T4 DNA ligase. This last step inserted an adapter sequence between the gene and vector—adding a BamHI restriction site downstream of the HO-HxoDH gene—and produced a linear, not circularized, product. The product was digested with BamHI and a ∼1000 base pair fragment, corresponding to the HO-HxoDH gene with a sticky NcoI 5' end as well as a sticky BamHI 3' end, was gel purified. More pET19b was digested with NcoI and BamHI, treated with shrimp alkaline phosphatase and a ∼5000 base pair fragment was gel purified. Finally, these two fragments were ligated to make pHicHis.

The plasmid was transformed into *E. coli* strain DH10B (Invitrogen, Carlsbad, CA) by electroporation with a Bio-Rad (Hercules, CA) GenePulser. DNA plasmid purification was done with a Wizard Midiprep kit from Promega (Madison, WI). The product was confirmed by DNA sequencing performed by Elim Biopharmaceuticals (Hayward, CA).

### Kinetic assays and data fitting

AATase activity was measured by MDH-coupled assays^32^ containing 200 m*M* TAPS, pH 8.0, 100 m*M* KCl, 150 μ*M* NADH, and 10 μ*M* PLP. Aspartate and αKG concentrations were varied. TATase activity was measured by HO-HxoDH-coupled assay^33^ containing 100 m*M* TAPS pH 8.0, 100 m*M* KCl, 150 μ*M* NADH, and 10 μ*M* PLP, while concentrations of Phe and αKG were varied. Activity with isoleucine, leucine, tyrosine and valine as substrates were measured with the same coupled assay. The rates of product formation were measured by loss of NADH absorbance at 340 nm. All measurements were made on an Agilent 8453 UV-Vis spectrophotometer or SpectraMax 190 UV-Vis plate-reader (Molecular Devices).

Kinetic data were fit with either the SAS (SAS Institute, Cary, NC) or Origin applications (OriginLab, Northampton, MA) to Eq. (1) describing a ping-pong bi-bi reaction:^34^


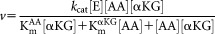
(1)

where [E] and [AA] are the concentrations of enzyme and amino acid substrate, respectively. Equation (1) reduces to:


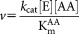
(2)

where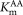 >> [AA]. Equation (2) was used to fit the data when saturating concentrations of amino acids could not be attained.

### Manual selection of aminotransferases

UniProt^35^ was queried for all sequences containing the keyword “aminotransferase” (1726 entries, as of April, 2003). The sequence alignment software, SATCHMO, was designed to align sequences with low pairwise similarity as well as those with higher overall sequence similarity but local variance in sequence.^36^ As pairwise similarity increases and local variance decreases, SATCHMO's alignment improves. However, it has a built-in limitation on the memory requirements for alignment, which, in practice, meant that only about 50 divergent aminotransferase sequences could be aligned by SATCHMO at a time. Therefore, the 1726 aminotransferase sequences were arbitrarily divided into 32 batches, each containing approximately 50 sequences.

In order to identify aminotransferases that were likely to be in the Iα subfamily, all sequence batches were iteratively aligned to each other and to two subfamily Iα reference sequences, cPigAAT and eAAT, with SATCHMO (note that cPigAAT and eAAT aligned well with each other as determined by visual inspection). Sequences were eliminated if they did not contain a lysine that aligned to the active site lysine of cPigAAT (K258*) according to SATCHMO's indication of alignable columns or if the alignment failed to converge (10 batches). This first round eliminated > 80 % of the sequences, leaving 325 sequences aligning with K258 of cPigAAT. These 325 sequences were arbitrarily divided into seven smaller batches and aligned under the same criteria, eliminating an additional 83 sequences. A third round was completed as a single batch with 242 remaining sequences and with the *minaff* option set to −0.5 because the method failed to converge with the default setting due to sequence divergence; 53 sequences were eliminated in this round. Analysis of the Swiss-Prot annotations and corresponding primary literature of the remaining 189 sequences revealed that all known subfamily Iα aminotransferases were localized to a distinct clade of 92 sequences in the tree produced by SATCHMO. The final SATCHMO alignment of these 92 subfamily Iα sequences was manually refined based on a structural alignment produced by MAPS^37^ of PDB entries 1AJS (cPigAAT), 2CST (cChickAAT), 1ASM (eAAT), 1MAP (mChickAAT), 3TAT (eTAT), 1AY5 (PdTAT), and 1YAA (SccAT).

This alignment of 92 sequences was used as the foundation for selecting a group of divergent proteins for kinetic characterization. Briefly, the sequences were grouped according to their similarity near the active site, and then a representative enzyme from each group was selected for further study. The unliganded eAAT crystal structure (PDB code 1ASN) was used to identify residues near the active site, defined here as being <15 Å from the nearest atom of the PLP cofactor. Moderate variability was determined from the overall percent conservation at a given position observed in the SATCHMO alignment of 92 sequences. For the purposes of this study, a residue has moderate variability if it is the same amino acid in at least 25%, but fewer than 75%, of the aligned sequences. Seventy-six positions out of ∼400 met the distance and variability (D&V) criteria, which we defined as <15 Å from cofactor and 25 to 75% identity. Each of the 92 subfamily Iα sequences in the SATCHMO alignment was compared with the set of 10 kinetically characterized reference sequences at each of these 76 positions. The latter reference set includes: (1) the proteins listed in Table I, which is a comprehensive set of class Iα aminotransferases for which there exists published kinetic data for aspartate and at least one of the aromatic substrates; (2) *Saccharomyces cerevisiae* cytosolic aspartate aminotransferase (SccAT), which has a published crystal structure; and (3) *P. aeruginosa* aspartate aminotransferase (PaAT).

**Table I:** Percent Sequence Identities of Aminotransferases with Known Substrate Specificities^a^

	AATases				TATases			
		
	cChickAAT	mChickAAT	cPigAAT	eAAT	eTAT	PdTAT	PhhC	SmTAT
AATases								
cChickAAT	—	43	**83**	37	38	33	35	32
mChickAAT	**43**	—	**43**	37	37	32	35	31
cPigAAT	**83**	43	—	38	37	32	37	32
eAAT	37	37	38	—	42	44	**46**	41
TATases								
eTAT	38	37	37	42	—	38	**44**	37
PdTAT	33	32	32	44	38	—	43	**45**
PhhC	35	35	37	**46**	44	43	—	44
SmTAT	32	31	32	41	37	**45**	44	—

a The highest percent identity for each row of sequences is in bold, while the lowest is underlined. The enzymes were assigned according to whether they do or do not exhibit high preferences for aspartate compared with aromatic amino acids (see Fig. 5). See text for enzyme name abbreviations.

**Figure 2:**
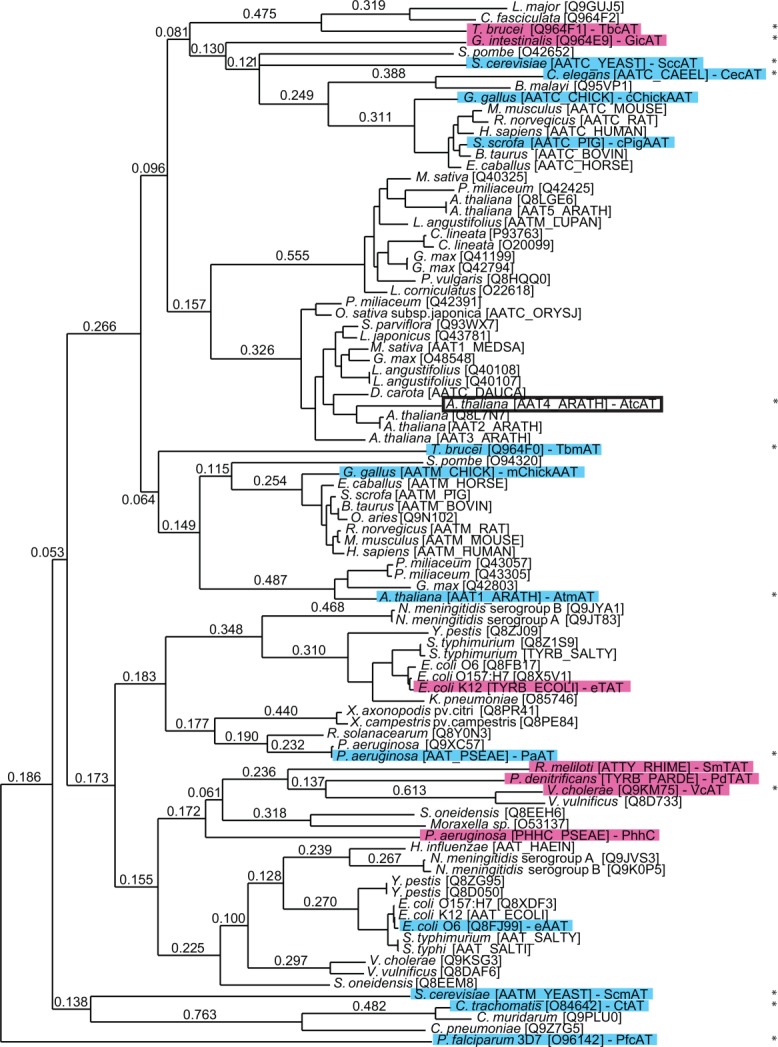
Groups of diverse aminotransferases. The choice of enzymes that were characterized (indicated by asterisks) and the grouping into similar sets by the D&V method are described in Materials and Methods. Identification numbers refer to Swiss-Prot entry names or UniProt accession numbers^38^ (UniProt accession numbers for Swiss-Prot sequences are provided in Supporting Information Table S1). The abbreviations used throughout this manuscript are as follows: AtcAT: AAT4_ARATH (*Arabidopsis thaliana* cytosolic); AtmAT: AAT1_ARATH (*A. thaliana* mitochondrial); CecAT: AATC_CAEEL (*C. elegans* cytosolic); CtAT: O84642 (*Chlamydia trachomatis*); GicAT: Q964E9 (*Giardia intestinalis* cytosolic); PfcAT: O96142 (*Plasmodium falciparum* cytosolic); ScmAT: AATM_YEAST (*Saccharomyces cerevisiae* mitochondrial); TbcAT: Q964F1 (*Trypanosoma brucei* cytosolic); TbmAT: Q964E0 (*T. brucei* mitochondrial); VcAT: Q9KM75 (*Vibrio cholerae*).

For each position chosen using the D&V criteria, a sequence's score (the D&V score) increased by one for each residue that was different from the corresponding residues in all the 10 characterized reference sequences in the given alignment. The total possible D&V score was 76, based on the total number of chosen residues in these sequences. Most of the sequences were similar or identical to those that had been previously characterized and consequently had D&V scores of 10 or less. A smaller set of thirty-two sequences with a D&V score > 10, and, therefore, greater sequence diversity near the active site, were carried forward for further analysis. A pair-wise score was calculated for each of these 32 top-scoring sequences to create a distance matrix in order to group the sequences according to their relative divergence. To compute the pair-wise score, two sequences were compared at each position that contributed to the original D&V score in that sequence, and one was added to the pair-wise score for each residue that was mismatched between the two sequences. These pair-wise scores are not necessarily symmetric since the positions contributing to the original D&V score may be different for each sequence. Pairs of sequences where both members of the pair score <9 relative to each other were placed into the same group (Fig. 2). One enzyme from each of these 10 groups was chosen for characterization based on gene availability. Thus, the active site of each enzyme that was selected was different from the 10 original reference enzymes, and also different from each of the other nine newly selected enzymes.

Using the D&V scores, we selected 10 distantly related aminotransferases that were previously uncharacterized to subject to kinetic analysis. As reported previously, attempts to obtain pure *Arabidopsis thaliana* cytosolic aminotransferase (AtcAT) were unsuccessful^31^ and the enzyme could not be characterized, but is included in the phylogenetic analyses here. The yeast cytosolic aminotransferase was also characterized because, while its crystal structure was solved,^15^ there are no reports in the literature of its kinetic activity with aromatic substrates.^39^ Kinetic data are also presented for the first time for PaAT, bringing the total number of Iα aminotransferases characterized here to 11.

### Phylogenetic analysis: The SIFTER method

**Figure 3:**
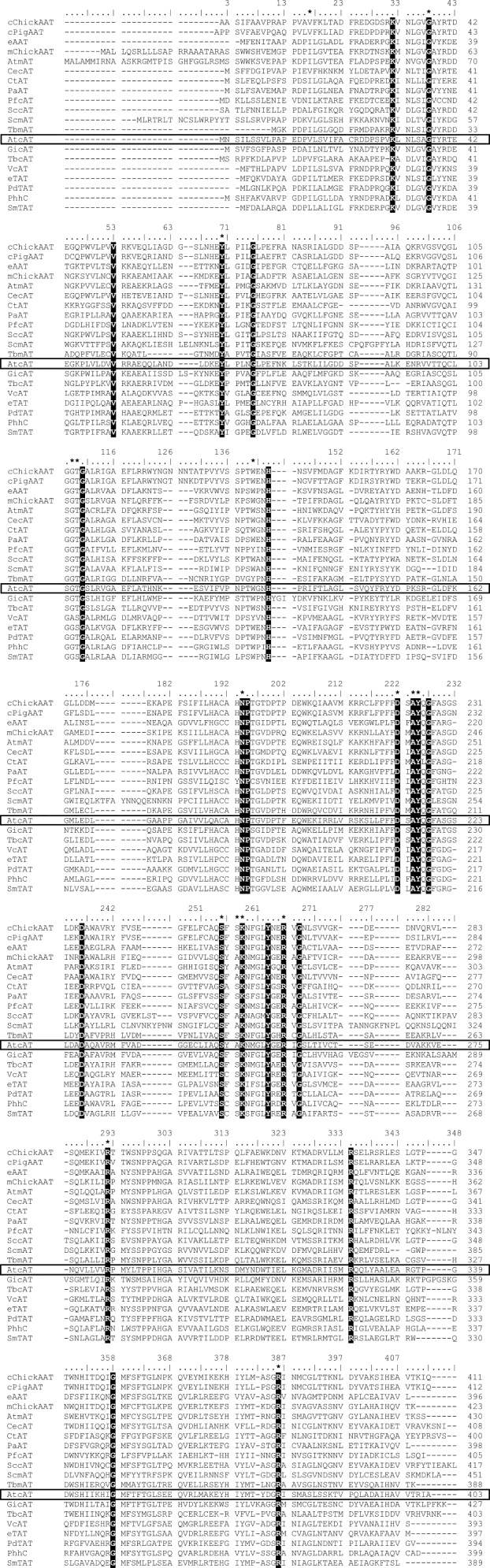
Dendrogram of subfamily Iα aminotransferases. The rooted tree of Iα aminotransferases was created with RAxML and the Consense application in the Phylip package using Iγ aminotransferases for the outgroup (outgroup not shown in figure for brevity). Branch length values are indicated on branches, but are omitted from select branches for clarity. The species and UniProt identifiers are indicated on each leaf (UniProt accession numbers corresponding to the Swiss-Prot sequences are in Supporting Information Table S1). Confirmed AATase and TATase annotations are highlighted in cyan and magenta, respectively, and AtcAT, for which kinetic data was not successfully obtained, is outlined in black. The 11 enzymes that were kinetically characterized in this work are indicated by an asterisk (*) to the right of the leaves.

The Statistical Inference of Function Through Evolutionary Relationships (SIFTER) method^40^ was applied to the aminotransferase Iα subfamily. We reconstructed a phylogenetic tree of the 92 Iα sequences identified using the iterative SATCHMO alignment method above. The 92 Iα sequences were aligned to 41 Iγ sequences with MUSCLE^41^ and manually reconciled to the structural alignment described above (MAPS^37^ alignment of seven Iα structures). A phylogeny was built from this alignment with RAxML, a fast, maximum likelihood method for reconstructing phylogenies,^42^,^43^ with 100 iterations of bootstrapping. The Iγ sequences were used as outgroup references to ensure proper rooting of the tree. A final consensus tree was created by the Consense program from the Phylip package with rooted trees.^44^ The subfamily phylogeny is shown in Figure 3.

We ran SIFTER 2.0^45^ on the phylogeny of 92 proteins belonging to the Iα subfamily in two ways: either including as input to SIFTER the existing set of eight functional characterizations (Table I), or including the 19 existing and new functional characterizations. In both cases, SIFTER produced a set of molecular function predictions for the proteins that did not have functional annotations as input. These results were used to perform a phylogenetic analysis of the family, and to compare phylogenetic analyses before and after the addition of the new functional annotations. We also performed leave-one-out cross-validation for both the existing set of functional characterizations and the existing and new functional characterizations to determine how the additional data improved predictions for uncharacterized proteins in this family. Leave-one-out cross-validation removes a single protein's experimental annotation and then predicts the annotation for that protein using only the remaining annotations.

## RESULTS

### Aminotransferase identification and alignment

The motivation for this research grew from three related goals: (1) to facilitate the prediction of function of uncharacterized aminotransferases from the Iα subfamily, (2) to identify the substrate specificity determinants, or the residues in the active site that play major roles in specificity and (3) to identify where and how substrate specificity is determined in the evolutionary history of this family using a phylogenetic analysis. The initial objective was then to gather substrate specificity data for a representative group of subfamily members to enable an informed phylogenetic analysis.

The construction of the set of broadly representative Iα aminotransferases was guided by the objective of obtaining a large cross-section of possible active sites that have AATase or TATase activity with the backbone of the Iα subfamily. A fingerprint of the conserved residues for this subfamily, which was defined by Jensen and Gu,^5^ was based on the limited set of Iα subfamily member protein sequences available before 1996. This fingerprint was not used to identify additional members of the subfamily in order to avoid bias against more distantly related members. Instead, we used the following alignment-based procedure to gather diverse members of this subfamily.

**Figure 4:**
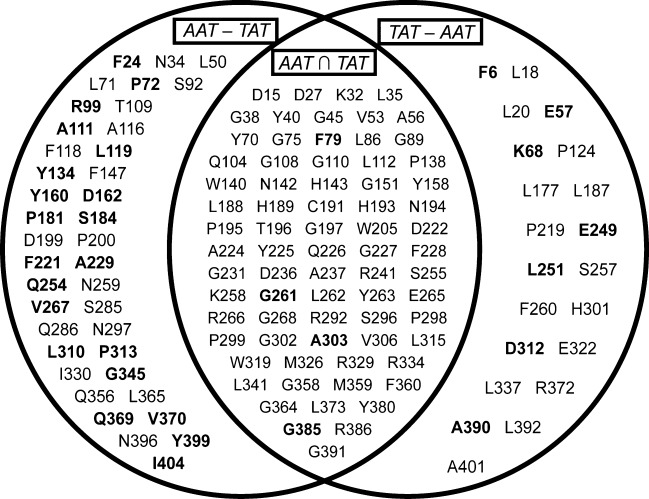
Sequence alignment of subfamily Iα aminotransferases. The kinetic parameters for the AATases, coded by the top four sequences, were determined earlier, and the bottom four are characterized TATases. The substrate specificities and kinetics of the remaining 12 enzymes were determined in this study. The sequences are ordered alphabetically within each group. The boxed sequence, AtcAT, has unknown substrate specificity (see Materials and Methods). The sequences above the box are now assigned as AATases, and those below are TATases. The alignment numbering is based on cChickAAT. The sequences were aligned by MUSCLE,^41^ with manual refinement based on a structural alignment produced by MAPS.^37^ The 23 positions highlighted in black are completely conserved in subfamily Iα aminotransferases. This is a reduction from the 51 specified in Jensen and Gu.^5^ The 16 first-shell residues (≤3.4 Å from the cofactor or inhibitor) are marked with an asterisk.

The UniProt database^38^ contains Swiss-Prot, a manually curated database, and TrEMBL, which is a computer-generated compilation of other databases, including GenBank. Since the objective was to cover the breadth of protein sequence and function, not to gather the largest possible data set of sequences, the UniProt database was probed for probable aminotransferase sequences. The breadth of sequence and function coverage for full-length enzymes in the UniProt database is comparable to GenBank; Swiss-Prot contains citations that go beyond sequencing studies; and Swiss-Prot annotations are, overall, more accurate.^10^ A full-text keyword search of UniProt for entries for “aminotransferase” yielded 1736 sequences that are potentially members of all aminotransferase families (as of publication, close to 110,000 entries now contain this keyword, consistent with general growth trends of UniProt). This sequence set was manually pruned by comparison to the sequences of two known Iα aminotransferases, cPigAAT and eAAT, in order to identify the likely Iα aminotransferases. An alignment of a similarly distant set of 20 Iα aminotransferases (Fig. 4) illustrates that the subfamily sequences align well and, despite the fairly large number of amino acid substitutions, some highly conserved regions are maintained across the subfamily.

The most reliable family I aminotransferase identifier is the sequence location of the active site lysine. From the pruned set, 189 sequences aligned at this locus with the cPigAAT K258 in multiple rounds of batch alignment (see Materials and Methods for details). Analysis of the Swiss-Prot sequences and their positions in the dendrogram calculated by SATCHMO revealed separate clades for the Iα subfamily, the histidinol-phosphate aminotransferase subfamily (Iβ), the Iγ AATases and TATases, and alanine aminotransferases (Iδ). This result is consistent with prior phylogenetic characterizations of these subfamilies.^5^ The final subfamily Iα clade contains 92 sequences, not all which are unique (Fig. 3). For example, there are three nearly identical sequences from *E. coli*: Swiss-Prot ID AAT_ECOLI, and UniProt AC Q8XDF3 and Q8FJ99, two of which are probably either population variants or sequencing errors.

While the first shell of residues around the active site in aminotransferases makes important contacts with the substrate and cofactor, PLP, second and third shell residues have also been shown to play roles in substrate specificity.^26^,^28^,^46^ All residues are within 32.2 Å of a PLP atom in the unliganded eAAT structure (PDB code 1ASN), and those that are three shells away from the PLP are within 16.3 Å, while those that are four shells away are ≤22 Å from a PLP atom (i.e., approximately 5 Å per shell).

**Figure 5:**
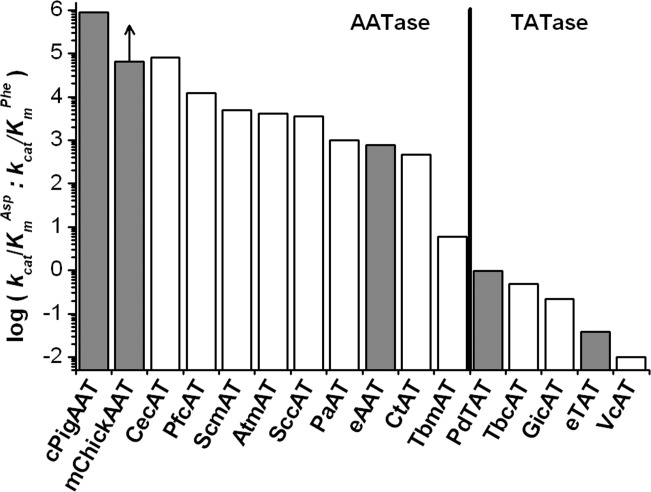
Venn diagram of conserved residues in AATases and TATases. Those conserved in ≥75% of the sequences for each substrate specificity were identified for the sequence alignment presented in Figure 4, excluding the uncharacterized sequence (AtcAT). Residues in bold type differ from the diagram presented in Rothman and Kirsch.^28^ Venn.out (written by Daniel Malashock, University of California, Berkeley, not published) was used to perform the sequence analysis to generate this figure.

To quantify the conservation of amino acids around the active site, we collected the set of sixteen amino acids that are ≤3.40 Å from the PLP (cofactor) or maleate (ligand) in the eAAT structure (PDB code 1ASM). The 16 residues in this first shell are: Ile17, Gly38, Tyr70, Gly108, Thr109, Trp140, Asn194, Asp222, Ala224, Tyr225, Ser255, Ser257, Lys258, Arg266, Arg292, and Arg386 (shown in Figs. 2 and 5). The quality score, or q-score, in the ClustalX alignment software^47^ for each of these columns denotes the level of similarity within that column of the alignment, with a value of 100 meaning that the amino acid is completely conserved across all of the sequences and a value of 0 indicating that the amino acid is not conserved at all. The sum of the *q*-scores for these 16 active site residues was 1446 (1600 maximum) using the alignment shown in Figure 4. To check whether the amino acids involved in the binding site are conserved relative to the remaining amino acids in this protein, we performed a permutation test by sampling randomly without replacement from all the columns in the alignment for which there was not a gap in the eAAT sequence. This test yields a significant *P* value (<10^−5^) indicating that the residues near the active site are significantly more conserved than residues chosen at random in this alignment. In particular, while the sum of the *q*-scores of these 16 columns in the alignment is 1446, the largest *q*-score sum of 16 columns randomly sampled without replacement 100,000 times was 1107. This level of conservation relative to overall sequence conservation in this family of proteins implies that these 16 amino acids are important for aminotransferase function. The results from the permutation test and the observations specific to the aminotransferase subfamily suggest that residues that are moderately conserved and near the active site are most likely to play key roles in substrate specificity.^28^,^48^

A goal of using the D&V scoring method was to select 10 new aminotransferases to characterize, effectively doubling the kinetic data for this subfamily. As described in Materials and Methods, all identified Iα aminotransferases (92 sequences) were compared with a set of 10 kinetically characterized reference aminotransferases at each of the 76 residues selected based on the distance and variability (D&V) criteria (see Materials and Methods for details). If overall sequence identity had been used as the selection criterion instead of a D&V method, a cut-off of 65% identity would have selected 12 sequences, in which each sequence is <65% identical to all of the kinetically characterized reference sequences and also <65% identical to each of the other 11 new sequences. In this scenario, while Groups 1, 3, 5, and 7 to 10 in Figure 2 would each be represented with one sequence and Group 2 with two sequences, Groups 4 and 6 would be eliminated and therefore no plant cytosolic or mitochondrial enzymes would have been chosen for characterization. The remaining 3 of these 12 sequences received low scores by the D&V method (a low score means high similarity to the reference set of sequences). *Schizosaccharomyces pombe* O94320 is the least similar of the three to the reference aminotransferases with a D&V score of 9 out of 76, while the other two are quite similar to the previously characterized set: their scores are both 4.

### Kinetic characterization

The kinetic constants characterizing the transamination of aspartate and phenylalanine for 11 aminotransferases, as compared with a representative AATase and TATase, are presented in Table II. *Caenorhabditis elegans* cytosolic AATase (CecAT) displays the strongest preference yet demonstrated for aspartate, with a specificity constant (*k*_cat_/*K*_m_) ratio of aspartate to phenylalanine of 80,000. Most enzymes with a preference for aspartate (*A. thaliana* mitochondrial AATase (AtmAT), CecAT, *Chlamydia trachomatis* AATase (CtAT), *Plasmodium falciparum* cytosolic AATase (PfcAT), PaAT, SccAT, and *S. cerevisiae* mitochondrial AATase (ScmAT)) have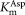 values of about 1 to 3 m*M*, and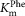 values >30 m*M*. The exception is *Trypanosoma brucei* mitochondrial AATase (TbmAT), which is a poor aminotransferase with high *K*_m_ values for all tested substrates (Asp, Phe, and Tyr). The kinetic constants for the transamination of tyrosine are comparable to those for phenylalanine for each of the four tested enzymes: *Giardia intestinalis* cytosolic TATase (GicAT), PfcAT, *T. brucei* cytosolic TATase (TbcAT) and TbmAT (data not shown).

**Table II:** Kinetic Constants for Newly Characterized Subfamily Iα Aminotransferases^a^

	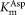 (m*M*)	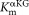 (m*M*)	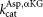 (s^−1^)	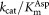 (M^−1^ s^−1^)	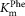 (m*M*)	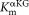 (m*M*)	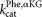 (s^−1^)	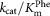 (M^−1^ s^−1^)	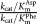
eAAT	1.75^24^	0.48^24^	159^24^	90,800^24^	NS^b^		NS	119^23^	760
eTAT^25^	3.8	0.80	140	37,000	0.26	1.7	250	960,000	0.038
AtmAT	2.5	2.2	89	36,000	NS^24^		NS	8.8	4,100
	(0.2)	(0.2)	(3)	(3,000)				(0.3)	(400)
CecAT	1.3	0.25	45	34,000	NS		NS	0.45	80,000
	(0.1)	(0.02)	(2)	(4,000)				(0.05)	(10,000)
CtAT	2.3	0.58	86	37,000	NS		NS	80	470
	(0.1)	(0.04)	(3)	(2,000)				(5)	(40)
GicAT	9.0	0.35	93	10,200	2.04	0.48	97	47000	0.22
	(0.5)	(0.02)	(3)	(600)	(0.08)	(0.02)	(2)	(2000)	(0.02)
PfcAT	1.0	0.8	36	35,000	NS		NS	3.0	12,000
	(0.1)	(0.1)	(3)	(5,000)				(0.1)	(2,000)
PaAT	2.01	2.8	99	47,000	NS		NS	47	1,000
	(0.2)	(0.3)	(4)	(5,000)				(3)	(100)
SccAT	2.7	1.2	168	63,000	NS		NS	18	3,600
	(0.2)	(0.1)	(8)	(6,000)				(1)	(400)
ScmAT	1.3	1.6	18	14,000	NS		NS	3.1	5,000
	(0.2)	(0.3)	(1)	(2,000)				(0.4)	(1,000)
TbcAT	9.6	0.54	105	11,000	5.3	0.84	118	22000	0.49
	(0.8)	(0.05)	(4)	(1,000)	(0.3)	(0.04)	(4)	(1000)	(0.05)
TbmAT	NS		NS	132	NS		NS	22.0	6.0
				(4)				(0.2)	(0.2)
VcAT	34	1.60	22.9	680	0.68	4.1	46	67,000	0.010
	(2)	(0.09)	(0.7)	(40)	(0.04)	(0.2)	(2)	(5,000)	(0.001)

a Conditions: pH 8.0 in 200 m*M* TAPS buffer and 100 m*M* KCl at 25°C, except TbmAT assays were done at an ionic strength = 0.43. Standard errors are in parentheses.

b NS, no saturation was observed with 40 m*M* of the specified amino acid substrate. *k*_cat_/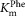 was determined with constant [αKG] >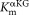 for the aspartate reaction. The [αKG] = 10 m*M* for the TbmAT assays.

All the enzymes, including the three with preferences for phenylalanine over aspartate (GicAT, TbcAT, and *Vibrio cholerae* TATase (VcAT)), exhibit low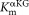 values (<3 m*M*). These three have values of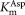 >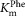, which accounts for most of the effect on the specificity ratios. VcAT has the lowest specificity ratio (0.010).

### Sequence similarities and differences

About half of the 51 positions that Jensen and Gu identified as invariant in Iα aminotransferases^5^ remain conserved in the aminotransferases characterized to date (Fig. 4). Some of the invariant residues described by them, which are not conserved in the alignment of the set of sequences used here, can be explained by conservative substitutions found in this expanded set of sequences. For example, residue 140, which forms a key interaction with the pyridine ring of PLP, is either the expected tryptophan or is a tyrosine. A subset of these twenty-three completely conserved residues located between residues 194 and 386 was used for a fingerprint search of the nonredundant sequence database with the BLAST program Seedtop (available from NCBI). Conservative, infrequent substitutions are also found in the full alignment of the resulting 2635 aminotransferase sequences for key positions such as 140.

The average distance of the closest atom of the twenty-three conserved residues from the most proximal atom of PLP (based on the eAAT structure 1ASN) is 7.3 Å; compared with an overall average distance of 16.1 Å (nearest atom to nearest atom) for all residues. Ten of these conserved residues are in direct contact with either PLP or with the ligand, maleate (based on the complexed structure 1ASM), and 15 are within the first two shells of active site residues (Fig. 1). Six of the conserved amino acids are glycine, and an additional six are conserved either as lysine or as arginine. These numbers are greater than what is observed among other sets of orthologous proteins in the three primary lineages: 26.0% of glycines are conserved in the active site of aminotransferases versus 13.2% glycine conservation overall; 8.7% and 17% of lysines and arginines, respectively, conserved in aminotransferase active sites versus 8.0% and 7.1% for each overall.^49^ While the functions of most of the lysines and arginines in the aminotransferases are known,^50^–^52^ the roles of the glycines (which are probably structural) and of many of the other conserved residues are not. It would seem productive to probe the small set of remaining conserved residues by mutagenesis in order to define further the mechanistic and structural characteristics of this group of enzymes.

The Venn diagram of Figure 5 shows that 71 residues are conserved in at least 15 of the now characterized sequences (Set *AAT ∩ TAT*), 39 are conserved exclusively in at least 9 of the AATases (Set *AAT–TAT*), and 21 are conserved in at least 6 of the TATases (Set *TAT–AAT*; i.e. at the ≥75% level of conservation). Thus, conservation among the residues common to both substrate specificities is greater than the conservation of residues common to either one of the substrate specificities. The phylogenetic-based analysis described below is in accord with these observations.

Using a less diverse set of aminotransferases, with a smaller percentage that had been kinetically characterized, Rothman and Kirsch earlier found that the putative AATases are more similar to the putative TATases than they are to other AATases, and vice versa,^28^ consistent with our observations from our more diverse set. However, they observed a nearly equivalent number of conserved residues with either specificity (|*AAT*−*TAT*| ≈ |*TAT*−*AAT*|), while our new set shows that the AATases are more similar to each other than the TATases are to each other (|*AAT*−*TAT*| = 39 as compared with |*TAT*−*AAT*| = 21 conserved amino acids). While the intersection of the sets in Rothman and Kirsch^28^ is quite similar to what is presented in Figure 5, the *AAT*−*TAT* and *TAT*−*AAT* sets are not. The magnitude of these differences is expected given the different amounts of substrate specificity data available for each of the two analyses. However, until we are closer to discovering the mechanism of substrate preference, it is difficult to speculate on relative similarity based on the sparse available data.

### Protein function prediction

We ran SIFTER on the phylogenetic tree for the aminotransferase Iα family containing 92 sequences (see Materials and Methods). In all experiments on this family, there were exactly two candidate functions from the Gene Ontology:^53^ L-aspartate:2-oxoglutarate aminotransferase activity (GO:0004069) and L-tyrosine:2-oxoglutarate aminotransferase activity (GO:0004838), corresponding to AATase and TATase activity, respectively. Using the default fixed parameters for SIFTER, we performed leave-one-out cross validation, including only the eight experimental annotations known before the experiments discussed here (those listed in Table I). SIFTER achieved 82% accuracy (9 of 11 substrate specificities were correct) in predicting the substrate preference of the newly evaluated enzymes; the substrate specificities of 2 of the 11 subsequently characterized sequences were predicted incorrectly (GicAT and TbcAT). We also performed leave-one-out cross-validation with the eight existing annotations plus the 11 additionally characterized proteins, for a total of 19 proteins, using the default SIFTER parameters, in order to determine if the additional characterizations improved prediction accuracy in this protein family. The additional data increased the accuracy slightly to 84% accuracy (16 of 19 correct substrate specificity predictions).

SIFTER and other methods for phylogenetic-based prediction of protein molecular function make the assumption that sequences that are closer in a phylogeny will tend to have more closely related substrate specificity.^54^ We see that this assumption is violated in this family: Figure 3 shows that there are at least three (or possibly more) locations in the phylogeny where the substrate specificity independently mutated to include aromatic amino acids. As a result, TATases appear to cluster in distant tree clades, and prediction accuracy is negatively impacted. Furthermore, additional data does not improve predictions substantially, as this assumption is violated in the newly characterized proteins according to this phylogeny just as in the previously characterized proteins.

A more sophisticated protein function prediction method might recognize this as convergent evolution and reduce the confidence values for predictions of specificities that arise independently in multiple places in the phylogeny. SIFTER includes functionality to estimate model parameters, including relative rate of convergent evolution; however, given the small number of observations and the number of parameters to estimate, we did not estimate model parameters in this application. Further, overlaying the relative activity numbers onto the phylogenetic tree does not improve the predictive power: the *k*_cat_/*K*_m_ specificity ratios for Asp:Phe do not cluster within the phylogeny (data not shown). Another possible route is to consider relevant motifs instead of the full sequence in the phylogenetic framework to improve prediction.

## DISCUSSION

### Selection of diverse enzymes

Enzymes were selected for characterization with the intended goal of finding those with divergent substrate specificity constants. The manual scoring algorithm presented here weighs sequence differences among the active site residues more heavily than those outside the active site in order to identify mutations that may have led to changes in substrate specificity (see description of D&V scoring in Materials and Methods.).

Table I lists the pairwise sequence identities of all the aminotransferases for which a definitive substrate specificity can be assigned. It is apparent from this table that overall sequence identity is not a reliable indicator of substrate specificity for aminotransferases. Choosing enzymes to characterize based on low overall sequence identity to the reference set would yield a collection with more variability in overall structure than in active site structure and substrate specificity. Within a family of proteins, those with the lowest overall sequence identity may simply have different folding or solubility requirements.

Table I indicates that AATase to TATase or vice versa specificity switching may have happened repeatedly in the evolution of the aminotransferase family. Two enzymes with high overall sequence identity may have different substrate specificities; thus, differences in active site residues should be weighed more heavily in the selection of new aminotransferases for characterization.

Experimental data suggesting that certain positions in the sequence are important for substrate specificity have also been unable to aid in the prediction of specificity in homologs. For example, most of the positions that were mutated in HEX are not conserved in AATase or TATase homologs that have since been characterized.^2^,^55^ Table III lists the differences at these six positions. With few exceptions, the amino acids observed in the homologs are not what the HEX experiments predict. Other research suggests that important specificity determining residues are more highly conserved in AATases than in TATases.^28^ In comparison with overall sequence identity criteria, the D&V method focused our selection on differences in the active site residues, which we argue are more likely to define the enzyme's substrate specificity in this subfamily.

**Table III:** Comparison of HEX Residues in Characterized Aminotransferases^a^

		TATases			AATases

Position	HEX mutation^b^	PdTAT^c^	SmTAT	PhhC	Eukaryotic^d^
39	V→L	V	V	V	A
41	K→Y	K	K	K	R
47	T→I	T	T	T	P
69	N→L	T	A	S	E
109	T→S	T	**S**	T	**T**
297	N→S	F	M	T	**N**

a Underlined amino acids indicate that the identity is switched relative to what would be expected from the HEX construct. Amino acids in bold font are the same as that predicted from HEX.

b eAAT residue mutated to its analogue in eTAT.^26^

c The comparison of PdTAT to the HEX positions was reported in Okamoto et al.^18^

d cPigAAT, cChickAAT, and mChickAAT.

### Substrate specificity

**Figure 6:**
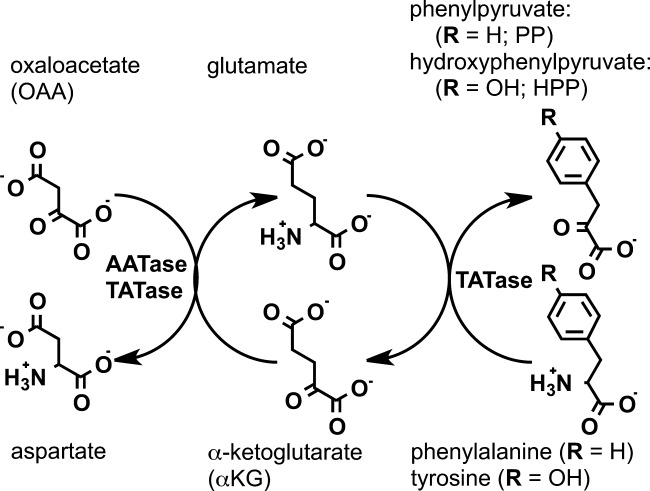
Ratios of specificity constants (*k*_cat_/*K*_m_) for transamination of aspartate versus phenylalanine for aminotransferases. Filled bars represent the data for previously characterized aminotransferases (cPigAAT,^56^ mChickAAT,^57^ eAAT,^24^ PdTAT,^55^ and eTAT^25^. The open bars provide the data for the 11 newly characterized enzymes from this investigation. The enzyme abbreviations are given in the text. An arrow indicates that only a lower-bound on the specificity ratio is available. The vertical line divides AATases from TATases; i.e., where the specificity ratio is greater than or less than 1.

Before this work, only three AATases and two TATases had been fully characterized kinetically. These AATases exhibit specificity ratios (*k*_cat_/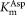
*k*_cat_/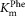) >900, while this value is <1 for the two TATases (Fig. 6). Thus the defining parameter for the specificity assignment was unambiguous. Five of the seven newly characterized AATases also exhibit specificity constants >900, but that for CtAT is 500 (Fig. 6 and Table II). TbmAT, although quite inactive (Table II), has a specificity ratio of only 6, and its specificity assignment is both more nuanced and less certain (see below). Two of the three novel TATases (TbcAT and GicAT) have specificity ratios between those of the previously characterized PdTAT and eTAT, while the specificity ratio for VcAT is the most discriminating class Iα aminotransferase for Phe over Asp yet found (specificity constant = 0.010). Thus the empirical criterion revealed by the limited set of available data continues to be valid. The same sharp drop in the specificity ratio separating the near continuum of AATase values from TATases (Fig. 6, vertical line) persists, except for that of TbmAT.

The TATases, GicAT, TbcAT and VcAT, have much higher *K*_m_ values for Asp than does either eTAT or PdTAT. The most striking example is VcAT, with a *K*_m_ of 34 m*M* for Asp, which is much higher than the intracellular Asp concentration of about 0.6 m*M* in *E. coli*.^58^ The distantly related family Iα TATases also have very high *K*_m_ values for Asp,^7^ but, unlike the enzymes characterized here, they have very low *k*_cat_ values for the Asp reaction.

There is significant variance in the data presented here relative to those reported in previous work.^59^ The kinetic data presented in Table II are for enzymes with C-terminal histidine tags.^31^ No saturation of aspartate up to 40 m*M* was observed here for C-terminal His_6_-tagged TbmAT, while Berger *et al*.^59^ report a *K*_m_ of 9.8 m*M* for N-terminal His_6_-tagged TbmAT. We find that C-terminal His_6_-tagged TbmAT is much less active than is GicAT, TbcAT or PfcAT and the *k*_cat_/*K*_m_ value for aspartate of 132 M^−1^ s^−1^ is fivefold smaller than that reported for N-terminal His_6_-tagged TbmAT.^59^ The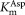 value for C-terminal His_6_-tagged PfcAT is 1.0 m*M* (this work) versus 5 m*M* for N-terminal His_6_-tagged PfcAT.^59^ The values for GicAT and TbcAT reported here agree with those in Berger *et al.*^59^ However, we find much higher *k*_cat_ values for aspartate transamination by GicAT, PfcAT, and TbcAT: 36 to 105 s^−1^ for C-terminal (Table II versus 3.2 to 6.4 s^−1^ for N-terminal^59^ tagged enzymes). This is additionally striking considering that the lower rate constants were obtained from measurements made at 37 C, while the present larger rate constants are found at 25 C. N-terminal affinity tags may have a deleterious effect on the activity of aminotransferases due to their proximity to the dimer interface and to the active site.^60^ Additionally, full ping-pong kinetics analyses were not carried out in the earlier study; thus, less accurate kinetic constants might have been obtained.

Results in Berger *et al.*^60^ show that N-terminal His_6_-tagged GicAT, PfcAT, TbcAT, and TbmAT transaminate several amino acids, suggesting that these enzymes exhibit very broad substrate specificity and function in methionine regeneration *in vivo*. A Iα aminotransferase from *Leishmania mexicana,* which has high sequence similarity to TbcAT, was also shown to transaminate methionine, aspartate, and phenylalanine, among other substrates.^61^ Malashock and Kirsch observed transamination of methionine by C-terminal His_6_-tagged GicAT (unpublished data) although recent specific activity data for C-terminal Strep-tagged PfcAT shows no activity toward methionine.^21^

The kinetic constants for aromatic amino acid transamination in Table II cannot be appropriately compared with the previous report as different co-substrates were used. Nonetheless, the present findings that GicAT and TbcAT are TATases are consistent with the overall conclusion in Berger *et al.*^59^ that these enzymes are broadly specific, and may play a key role in methionine recycling. The *in vivo* function of these aminotransferases is yet to be elucidated; therefore we tentatively classify them as TATases, in order to be consistent with the current nomenclature conventions. However, if the primary function of these proteins is found to be in methionine recycling, rather than aspartate or tyrosine/phenylalanine metabolism, this assignment should be revisited.

The major role of mitochondrial aminotransferases is in the malate-aspartate shuttle; therefore, they should exhibit strong preferences for aspartate and glutamate over other substrates. AtmAT, ScmAT, and TbmAT were annotated as mitochondrial enzymes because they have longer N-termini, as found for other nuclear-encoded aminotransferases from that organelle (i.e., mChickAAT vs. cChickAAT).^59^,^62^,^63^ Morin *et al.* noted that a signal sequence similar to that of ScmAT is present on the N-terminus of a mitochondrial alcohol dehydrogenase.^62^,^64^ Mitochondrial signal sequences are cleaved *in vivo*; therefore kinetic data collected on enzymes with intact N-termini may not reflect *in vivo* functionality. Nonetheless, the substrate specificity ratios should not be significantly affected by the presence of the signal sequence.

AtmAT and TbmAT were characterized with intact signal sequences, while ScmAT was characterized without its putative signal sequence. AtmAT and ScmAT have reasonable *k*_cat_ values for the aspartate reaction (89 s^−1^ and 18 s^−1^, respectively), while TbmAT exhibits low activity toward Asp, Phe, and Tyr (*k*_cat_/*K*_m_ values are from 22 to 132 M^−1^ s^−1^; tyrosine transamination data not shown). This *k*_cat_ value for AtmAT is about half of 205 s^−1^, the value that was previously published for N-terminally truncated AtmAT.^65^ Although the kinetics of truncated AtmAT were determined from linear regression at a single concentration of co-substrate, that alone cannot explain the large difference in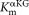 presented here (Table II; 2.2 m*M* vs. 0.26 m*M*).^65^ The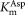 values are similar for the full-length and truncated forms (2.5 m*M* vs. 3.0 m*M*) and, consequently, the specificity constants for Asp for the two forms of AtmAT are within a factor of 2.

The specificity ratio of Asp to Phe for AtmAT is 4100 and for ScmAT is 5000, consistent with AATase annotations (i.e., >>1). In contrast, the specificity ratio of TbmAT is only 6; it is an AATase, but does not discriminate well between substrates. While AATases do have a well-known function in mitochondria, no function is known for a TATase in that organelle; thus, the lack of specificity of TbmAT for aspartate is unexpected. The mitochondrial signal sequence or lengthy purification process^31^ may be responsible for the low activity of TbmAT, but it is also possible that it is neither an AATase nor a TATase and that the true substrate has not been identified.

### Phylogenetic analysis

The set of 19 characterized enzymes, including the 11 presented here, are scattered throughout the subfamily Iα phylogeny (Fig. 3), even though the overall sequence identities and phylogeny were not considered in their selection. The previously characterized aminotransferases are localized to certain sections of the phylogeny, and the new characterizations fill in some, but not all, gaps.

The application of phylogenetic methods to protein function determination is predicated on the assumption that molecular function (including substrate specificity) evolves in parallel with sequence.^66^ This family shows more independent changes in substrate specificity than are suggested by the evolutionary distances. Thus, in order to localize those independent substrate preference mutations precisely within the phylogeny, it appears that more experimental data are needed, or alternative methods for protein function prediction are required. The current set of annotations shows that there are multiple instances of mutation in the tree, but there are insufficient characterizations to localize these mutations to a single branch. Multiple instances of independent, parallel evolution do not preclude a phylogenetic-based analysis.

Despite these frequent function changes, SIFTER predicted 16 of the 19 specificities correctly. With only the previously-known annotations, SIFTER predicted 9 of the 11 specificities correctly, so the analysis maintains a low cross-validation error rate with the additional protein characterizations. This number is indicative of how complete the current information is to predict substrate specificity of all of the remaining members of the tree and to enable localization of the substrate specificity mutation events. Thus, we can quantify the progress made in predicting substrate preference for uncharacterized proteins and also in localizing mutation events in the phylogeny with these characterizations.

A phylogenetic analysis suggests a series of hypotheses about this subfamily of proteins. SIFTER predicts that the root node of the phylogeny is an AATase, both before and after inclusion in the phylogeny of the set of proteins experimentally characterized here. This implies that the ancestral protein in this family may have had a preference for aspartate and that a preference for tyrosine is a more recent development, in agreement with prior analysis^5^,^28^ (although other research suggests that the ancestral enzyme had broad specificity that was subsequently narrowed^5^). The hypothesis that the ancestral Iα enzyme was an AATase is further supported by an analysis based on parsimony: if we assume that this phylogeny has the minimal number of changes in substrate preference (i.e., three), this is only possible given the current annotations and assuming the phylogeny is accurate when the protein at the root node is an AATase. If we consider TATase activity to be at the root of the tree and AATase activity independently became the dominant function, it would have required at least five separate instances of substrate preference switching to explain the current configuration. It is quite possible that additional enzyme characterizations will increase this number, because of the large number of remaining uncharacterized subfamily members.

Because of the diversity of organisms in this subfamily phylogeny, another hypothesis we can make is that a subfamily Iα AATase may have been present in the common ancestor of bacteria and eukaryotes (representatives from archaea are notably absent). It is possible that bacteria and eukaryotes both require at least one AATase as no major lineage shows evidence of a deletion of this enzyme. The AATases of higher organisms cluster well near the top of the phylogeny in Figure 3, with mitochondrial forms in the lower clades in this region, and we can see that the protozoan aminotransferases either cluster in the middle or are segregated from the bulk of the tree (e.g., PfcAT at the bottom of Fig. 3). A fingerprint search of the gene databases (based on the new sequence alignment in Fig. 4) supports the hypothesis that animals, plants, and fungi express at least two Iα AATases, corresponding to cytosolic and mitochondrial localization, with some plants and fungi expressing even more Iα aminotransferases as exemplified by *A. thaliana* and consistent with plant robustness and redundancy findings (data not shown). In contrast, protozoa may have only one Iα aminotransferase, either a cytosolic or mitochondrial AATase, and any other Iα aminotransferases may have broader substrate specificity. Finally, bacteria tend to have two Iα aminotransferases, representing each of the two specificities, although there are exceptions (e.g., the *C. trachomatis* genome encodes only one: CtAT (data not shown)). These same trends are observed in the more limited phylogeny presented in Figure 3.

## CONCLUSION

The subfamily Iα aminotransferases characterized here prove to be diverse evolutionarily and also in terms of substrate preferences. Phylogenetic analysis illustrates the complexity of the evolution and highlights the difficulty in predicting precise molecular function in this subfamily. However, additional data improves predictive capabilities in a protein family such as the Iα aminotransferases where substrate specificity changes occur repeatedly in the family's evolution. Additionally, further studies that build on the sequence, phylogenetic, and kinetic data presented here can be targeted to identify the cellular function of Iα aminotransferases as well as the role of particular conserved or variable residues in the subfamily. In particular, although our analysis indicates that the active sites of the plant enzymes are relatively conserved, the overall sequences are distinct from the other enzymes for which we now have activity data. We have shown here that closely related enzymes often have different specificities and the extent of such diversity in the other regions of the phylogeny remains an area for future study. Together with the continued deposition of aminotransferase crystal structures, the kinetics data presented here rejuvenates this subfamily for new insights into sequence-structure-function relationships.

## Footnotes

* Chicken cytosolic AATase numbering

## Abbreviations

αKG: α-ketoglutarate
AATase: aspartate aminotransferase
AtcAT, AtmAT, CecAT, CtAT, GicAT, PfcAT, ScmAT, TbcAT, TbmAT, and VcAT: aminotransferases (see Fig. 2 for sources)
cChickAAT: cytosolic chicken AATase
cPigAAT: cytosolic pig AATase
D&V: distance and variability selection method
eAAT: *E. coli* AATase
eTAT: *E. coli* TATase
HO-HxoDH: hydroxyisocaproate dehydrogenase
HPP: hydroxyphenylpyruvate
mChickAAT: mitochondrial chicken AATase
MDH: malate dehydrogenase
OAA: oxaloacetate
PaAT: *Pseudomonas aeruginosa* AATase
PdTAT: *Paracoccus denitrificans* TATase
PhhC: *P. aeruginosa* TATase
PLP: pyridoxal 5′-phosphate
PP: phenylpyruvate
SccAT: *S. cerevisiae* AATase
SIFTER: Statistical Inference of Function Through Evolutionary Relationships
TATase: tyrosine aminotransferase

## References

[1] Gelfand DH, Steinberg RA (1977). *Escherichia coli* mutants deficient in the aspartate and aromatic amino acid aminotransferases. J Bacteriol.

[2] Gu W, Song J, Bonner CA, Xie G, Jensen RA (1998). PhhC is an essential aminotransferase for aromatic amino acid catabolism in *Pseudomonas aeruginosa*. Microbiology.

[3] Finn RD, Mistry J, Tate J, Coggill P, Heger A, Pollington JE, Gavin OL, Gunasekaran P, Ceric G, Forslund K, Holm L, Sonnhammer EL, Eddy SR, Bateman A (2010). The Pfam protein families database. Nucleic Acids Res.

[4] Mehta PK, Hale TI, Christen P (1993). Aminotransferases: demonstration of homology and division into evolutionary subgroups. Eur J Biochem.

[5] Jensen RA, Gu W (1996). Evolutionary recruitment of biochemically specialized subdivisions of Family I within the protein superfamily of aminotransferases. J Bacteriol.

[6] Sivaraman S, Kirsch JF (2006). The narrow substrate specificity of human tyrosine aminotransferase—the enzyme deficient in tyrosinemia type II. FEBS J.

[7] Nowicki C, Hunter GR, Montemartini-Kalisz M, Blankenfeldt W, Hecht H, Kalisz HM (2001). Recombinant tyrosine aminotransferase from *Trypanosoma cruzi*: structural characterization and site directed mutagenesis of a broad substrate specificity enzyme. Biochim Biophys Acta.

[8] Brenner SE (1999). Errors in genome annotation. Trends Genet.

[9] Devos D, Valencia A (2001). Intrinsic errors in genome annotation. Trends Genet.

[10] Schnoes AM, Brown SD, Dodevski I, Babbitt PC (2009). Annotation error in public databases: misannotation of molecular function in enzyme superfamilies. PLoS Comput Biol.

[11] Dean CR, Franklund CV, Retief JD, Coyne J, M J, Hatano K, Evans DJ, Pier GB, Goldberg JB (1999). Characterization of the serogroup O11 O-antigen locus of *Pseudomonas aeruginosa* PA103. J Bacteriol.

[12] Zhao G, Xia T, Song J, Jensen RA (1994). *Pseudomonas aeruginosa* possesses homologues of mammalian phenylalanine hydroxylase and 4 alpha-carbinolamine dehydratase/DCoH as part of a three-component gene cluster. Proc Natl Acad Sci USA.

[13] DeLano WL (2002).

[14] Ford GC, Eichele G, Jansonius JN (1980). Three-dimensional structure of a pyridoxal-phosphate-dependent enzyme, mitochondrial aspartate aminotransferase. Proc Natl Acad Sci USA.

[15] Jeffery CJ, Barry T, Doonan S, Petsko GA, Ringe D (1998). Crystal structure of *Saccharomyces cerevisiae* cytosolic aspartate aminotransferase. Protein Sci.

[16] Kamitori S, Okamoto A, Hirotsu K, Higuchi T, Kuramitsu S, Kagamiyama H, Matsuura Y, Katsube Y (1990). Three-dimensional structures of aspartate aminotransferase from *Escherichia coli* and its mutant enzyme at 2.5 Å resolution. J Biochem (Tokyo).

[17] Malashkevich VN, Strokopytov BV, Borisov VV, Dauter Z, Wilson KS, Torchinsky YM (1995). Crystal structure of the closed form of chicken cytosolic aspartate aminotransferase at 1.9 Å resolution. J Mol Biol.

[18] Okamoto A, Nakai Y, Hayashi H, Hirotsu K, Kagamiyama H (1998). Crystal structures of *Paracoccus denitrificans* aromatic amino acid aminotransferase: a substrate recognition site constructed by rearrangement of hydrogen bond network. J Mol Biol.

[19] Rhee S, Silva MM, Hyde CC, Rogers PH, Metzler CM, Metzler DE, Arnone A (1997). Refinement and comparisons of the crystal structures of pig cytosolic aspartate aminotransferase and its complex with 2-methylaspartate. J Biol Chem.

[20] Han Q, Cai T, Tagle D, Li J (2010). Structure, expression, and function of kynurenine aminotransferases in human and rodent brains. Cell Mol Life Sci.

[21] Wrenger C, Müller IB, Schifferdecker AJ, Jain R, Jordanova R, Groves MR (2011). Specific inhibition of the aspartate aminotransferase of *Plasmodium falciparum*. J Mol Biol.

[22] Wilson CA, Kreychman J, Gerstein M (2000). Assessing annotation transfer for genomics: quantifying the relations between protein sequence, structure and function through traditional and probabilistic scores. J Mol Biol.

[23] Luong TN, Kirsch JF (2001). A general method for the quantitative analysis of functional chimeras: applications from site-directed mutagenesis and macromolecular association. Protein Sci.

[24] Gloss LM, Planas A, Kirsch JF (1992). Contribution to catalysis and stability of the five cysteines in *Escherichia coli* aspartate aminotransferase. Preparation and properties of a cysteine-free enzyme. Biochemistry.

[25] Hayashi H, Inoue K, Nagata T, Kuramitsu S, Kagamiyama H (1993). *Escherichia coli* aromatic amino acid aminotransferase: characterization and comparison with aspartate aminotransferase. Biochemistry.

[26] Onuffer JJ, Kirsch JF (1995). Redesign of the substrate specificity of *Escherichia coli* aspartate aminotransferase to that of *Escherichia coli* tyrosine aminotransferase by homology modeling and site-directed mutagenesis. Protein Sci.

[27] Shaffer WA, Luong TN, Rothman SC, Kirsch JF (2002). Quantitative chimeric analysis of six specificity determinants that differentiate *Escherichia coli* aspartate from tyrosine aminotransferase. Protein Sci.

[28] Rothman SC, Kirsch JF (2003). How does an enzyme evolved in vitro compare to naturally occurring homologs possessing the targeted function? Tyrosine aminotransferase from aspartate aminotransferase. J Mol Biol.

[29] Onuffer JJ, Kirsch JF (1994). Characterization of the apparent negative co-operativity induced in *Escherichia coli* aspartate aminotransferase by the replacement of Asp222 with alanine. Evidence for an extremely slow conformational change. Protein Eng.

[30] Aitken SM, Kim DH, Kirsch JF (2003). *Escherichia coli* cystathionine gamma-synthase does not obey ping-pong kinetics. Novel continuous assays for the elimination and substitution reactions. Biochemistry.

[31] Muratore KE, Srouji JR, Chow MA, Kirsch JF (2008). Recombinant expression of twelve evolutionarily diverse subfamily I[alpha] aminotransferases. Protein Expr Purif.

[32] Karmen A, Wroblewski F, Ladue JS (1955). Transaminase activity in human blood. J Clin Invest.

[33] Luong TN, Kirsch JF (1997). A continuous coupled spectrophotometric assay for tyrosine aminotransferase activity with aromatic and other nonpolar amino acids. Anal Biochem.

[34] Velick SF, Vavra J (1962). A kinetic and equilibrium analysis of the glutamic oxaloacetate transaminase mechanism. J Biol Chem.

[35] Boeckmann B, Bairoch A, Apweiler R, Blatter M-C, Estreicher A, Gasteiger E, Martin MJ, Michoud K, O′Donovan C, Phan I, Pilbout S, Schneider M (2003). The SWISS-PROT protein knowledgebase and its supplement TrEMBL in 2003. Nucleic Acids Res.

[36] Edgar RC, Sjolander K (2003). SATCHMO: sequence alignment and tree construction using hidden Markov models. Bioinformatics.

[37] Zhang Z, Lindstam M, Unge J, Peterson C, Lu G (2003). Potential for Dramatic Improvement in Sequence Alignment against Structures of Remote Homologous Proteins by Extracting Structural Information from Multiple Structure Alignment. J Mol Biol.

[38] Bairoch A, Apweiler R, Wu CH, Barker WC, Boeckmann B, Ferro S, Gasteiger E, Huang H, Lopez R, Magrane M, Martin MJ, Natale DA, O′Donovan C, Redaschi N, Yeh L-SL (2005). The Universal Protein Resource (UniProt). Nucleic Acids Res.

[39] Cronin VB, Maras B, Barra D, Doonan S (1991). The amino acid sequence of the aspartate aminotransferase from baker's yeast (*Saccharomyces cerevisiae*. Biochem J.

[40] Engelhardt BE, Jordan MI, Muratore KE, Brenner SE (2005). Protein molecular function prediction by bayesian phylogenomics. PLoS Comput Biol.

[41] Edgar RC (2004). MUSCLE: multiple sequence alignment with high accuracy and high throughput. Nucleic Acids Res.

[42] Stamatakis A (2006). RAxML-VI-HPC: maximum likelihood-based phylogenetic analyses with thousands of taxa and mixed models. Bioinformatics.

[43] Stamatakis A, Hoover P, Rougemont J (2008). A rapid bootstrap algorithm for the RAxML Web servers. Syst Biol.

[44] Felsenstein J (1997). An alternating least squares approach to inferring phylogenies from pairwise distances. Syst Biol.

[45] Engelhardt BE, Jordan MI, Srouji JR, Brenner SE (2011). Genome-scale phylogenetic function annotation of large and diverse protein families. Genome Res.

[46] Miyazawa K, Kawaguchi S, Okamoto A, Kato R, Ogawa T, Kuramitsu S (1994). Construction of aminotransferase chimeras and analysis of their substrate specificity. J Biochem (Tokyo).

[47] Thompson JD, Gibson TJ, Plewniak F, Jeanmougin F, Higgins DG (1997). The CLUSTAL_X windows interface: flexible strategies for multiple sequence alignment aided by quality analysis tools. Nucleic Acids Res.

[48] Yano T, Oue S, Kagamiyama H (1998). Directed evolution of an aspartate aminotransferase with new substrate specificities. Proc Natl Acad Sci USA.

[49] Brooks DJ, Fresco JR (2002). Increased frequency of cysteine, tyrosine, and phenylalanine residues since the last universal ancestor. Mol Cell Proteomics.

[50] Jansonius JN, Eichele G, Ford GC, Picot D, Thaller C, Vincent MG, Christen P, Metzler DE (1985). Spatial structure of mitochondrial aspartate aminotransferase. Transaminases, Vol. 2. Biochemistry.

[51] Sandmeier E, Christen P (1982). Chemical modification of a functional arginyl residue (Arg 292) of mitochondrial aspartate aminotransferase. Identification as the binding site for the distal carboxylate group of the substrate. J Biol Chem.

[52] Slebe JC, Martinez-Carrion M (1976). Carbamylation of aspartate transaminase and the pK value of the active site lysyl residue. J Biol Chem.

[53] Ashburner M, Ball CA, Blake JA, Botstein D, Butler H, Cherry JM, Davis AP, Dolinski K, Dwight SS, Eppig JT, Harris MA, Hill DP, Issel-Tarver L, Kasarskis A, Lewis S, Matese JC, Richardson JE, Ringwald M, Rubin GM, Sherlock G (2000). Gene ontology: tool for the unification of biology. The Gene Ontology Consortium. Nat Genet.

[54] Engelhardt BE, Stephens M (2010). Analysis of population structure: a unifying framework and novel methods based on sparse factor analysis. PLoS Genet.

[55] Oue S, Okamoto A, Nakai Y, Nakahira M, Shibatani T, Hayashi H, Kagamiyama H (1997). *Paracoccus denitrificans* aromatic amino acid aminotransferase: a model enzyme for the study of dual substrate recognition mechanism. J Biochem (Tokyo).

[56] Pan QW, Tanase S, Fukumoto Y, Nagashima F, Rhee S, Rogers PH, Arnone A, Morino Y (1993). Functional roles of valine 37 and glycine 38 in the mobile loop of porcine cytosolic aspartate aminotransferase. J Biol Chem.

[57] Pan P, Jaussi R, Gehring H, Giannattasio S, Christen P (1994). Shift in pH-rate profile and enhanced discrimination between dicarboxylic and aromatic substrates in mitochondrial aspartate aminotransferase Y70H. Biochemistry.

[58] Lowry OH, Carter J, Ward JB, Glaser L (1971). The effect of carbon and nitrogen sources on the level of metabolic intermediates in *Escherichia coli*. J Biol Chem.

[59] Berger LC, Wilson J, Wood P, Berger BJ (2001). Methionine regeneration and aspartate aminotransferase in parasitic protozoa. J Bacteriol.

[60] Fukumoto Y, Tanase S, Nagashima F, Ueda S, Ikegami K, Morino Y (1991). Structural and functional role of the amino-terminal region of porcine cytosolic aspartate aminotransferase. Catalytic and structural properties of enzyme derivatives truncated on the amino-terminal side. J Biol Chem.

[61] Vernal J, Cazzulo JJ, Nowicki C (1998). Isolation and partial characterization of a broad specificity aminotransferase from *Leishmania mexicana* promastigotes. Mol Biochem Parasitol.

[62] Morin PJ, Subramanian GS, Gilmore TD (1992). AAT1, a gene encoding a mitochondrial aspartate aminotransferase in *Saccharomyces cerevisiae*. Biochim Biophys Acta.

[63] Schultz CJ, Coruzzi GM (1995). The aspartate aminotransferase gene family of *Arabidopsis* encodes isoenzymes localized to three distinct subcellular compartments. Plant J.

[64] Young ET, Pilgrim D (1985). Isolation and DNA sequence of ADH3, a nuclear gene encoding the mitochondrial isozyme of alcohol dehydrogenase in *Saccharomyces cerevisiae*. Mol Cell Biol.

[65] Wilkie SE, Warren MJ (1998). Recombinant expression, purification, and characterization of three isoenzymes of aspartate aminotransferase from *Arabidopsis thaliana*. Protein Expr Purif.

[66] Eisen JA (1998). Phylogenomics: improving functional predictions for uncharacterized genes by evolutionary analysis. Genome Res.

