# Supplementary material for: Molecular function prediction for a family exhibiting evolutionary tendencies toward substrate specificity swapping: Recurrence of tyrosine aminotransferase activity in the Iα subfamily
Source: Proteins. 2013 May 13;81(9):1593–609. doi: 10.1002/prot.24318 (PMC3823064; doi:10.1002/prot.24318)
Supplement: Supplementary file 1 [file prot0081-1593-SD1.doc]

Table S1: UniProt accession numbers corresponding to Swiss-Prot entry names

| **Swiss-Prot ID** | **UniProt AC** |
| --- | --- |
| AAT_ECOLI | P00509 |
| AAT_HAEIN | P44425 |
| AAT_PSEAE | P72173 |
| AAT_SALTI | Q56114 |
| AAT_SALTY | P58661 |
| AAT1_ARATH | P46643 |
| AAT1_MEDSA | P28011 |
| AAT2_ARATH | P46645 |
| AAT3_ARATH | P46644 |
| AAT4_ARATH | P46646 |
| AAT5_ARATH | P46248 |
| AATC_BOVIN | P33097 |
| AATC_CAEEL | Q22067 |
| AATC_CHICK | P00504 |
| AATC_DAUCA | P28734 |
| AATC_HORSE | P08906 |
| AATC_HUMAN | P17174 |
| AATC_MOUSE | P05201 |
| AATC_ORYSA | P37833 |
| AATC_PIG | P00503 |
| AATC_RAT | P13221 |
| AATC_YEAST | P23542 |
| AATM_BOVIN | P12344 |
| AATM_CHICK | P00508 |
| AATM_HORSE | P08907 |
| AATM_HUMAN | P00505 |
| AATM_LUPAN | P26563 |
| AATM_MOUSE | P05202 |
| AATM_PIG | P00506 |
| AATM_RAT | P00507 |
| AATM_YEAST | Q01802 |
| ATTY_RHIME | Q02636 |
| PHHC_PSEAE | P43336 |
| TYRB_ECOLI | P04693 |
| TYRB_PARDE | P95468 |
| TYRB_SALTY | P74861 |
